# Structural basis for heme binding by the Shr protein from *Streptococcus pyogenes*

**DOI:** 10.1016/j.jbc.2025.111012

**Published:** 2025-12-05

**Authors:** Kanta Seki, Akinobu Senoo, Satoru Nagatoishi, Saeko Yanaka, Makoto Nakakido, Kouhei Tsumoto, Jose M.M. Caaveiro

**Affiliations:** 1 Laboratory of Protein Drug Discovery, Graduate School of Pharmaceutical Sciences, Kyushu University, Fukuoka, Japan; 2 Medical Device Development and Regulation Research Center, School of Engineering, The University of Tokyo, Bunkyo-ku, Tokyo, Japan; 3 Department of Bioengineering, School of Engineering, The University of Tokyo, Bunkyo-ku, Tokyo, Japan; 4 Materials and Structures Laboratory, Institute of Integrative Research, Institute of Science Tokyo, Yokohama, Kanagawa, Japan; 5 Department of Chemistry and Biotechnology, School of Engineering, The University of Tokyo, Bunkyo-ku, Tokyo, Japan

**Keywords:** X-ray crystallography, heme binding domain, heme transfer, Streptococcus pyogenes, axial coordination, heme, NEAT domain, Shrs

## Abstract

*Streptococcus pyogenes* causes a range of infectious diseases. In an era of increasing antibiotic resistance, new antimicrobial strategies targeting virulence factors, rather than essential survival mechanisms, are being explored. A key virulence factor in *S*. *pyogenes* is the bacterial iron acquisition system, because iron is essential but limited in the host due to sequestration by proteins like hemoglobin. The bacteria *S*. *pyogenes* possesses the Shr protein that acquires heme from host hemoglobin and transfers it to Shp, a membrane proximity protein. Shr comprises multiple domains, including two NEAr-Transporter (NEAT) domains that directly bind to heme. While structural information of NEAT domains from other bacteria are available, the structure of NEAT domains from Shr remains unknown. In this study, crystal structures of Linker-NEAT1 and NEAT2 domains were determined to 2.35 Å resolution and 2.66 Å resolution, respectively. Structural and mutational analyses revealed that methionine residues play a key role in heme binding, which seems to be a characteristic of heme-binding proteins from *S*. *pyogenes*, but not of NEAT domains from other gram-positive species. These findings enhance our understanding of heme acquisition in *S*. *pyogenes* and may guide novel therapeutic approaches.

*Streptococcus pyogenes*, also known as Group A *Streptococcus* (GAS), is a gram-positive bacterium that causes a wide range of pathologies, from mild cases such as sore throat and pharyngitis to severe cases such as streptococcal toxic shock syndrome and necrotizing fasciitis (1). Currently, infections caused by *S*. *pyogenes* are mainly treated with antibiotics, although strains showing resistance to several antibiotics, including macrolides and fluoroquinolones, have been reported (2, 3). Under such circumstances, there is an urgent demand to develop antimicrobial agents, especially those that target routes that are less prone to induce resistance. Attempts are being made to develop drugs that target virulence factors necessary only for the bacterial infection to the host, rather than acting on factors essential for bacterial survival and thus reducing the appearance of resistant strains (4). Therefore, functional analysis of proteins involved in infection as virulence factors is becoming increasingly important.

One of the virulence factors that has attracted attention as an antimicrobial target is the iron acquisition system of bacteria (5). Iron is an essential nutrient for all organisms, including pathogenic bacteria, because it is one of the central metals that functions as an electron carrier and cofactor for a vast number of proteins (6). In living organisms, the concentration of iron in solution is extremely low because it is trapped by iron-binding proteins such as hemoglobin, myoglobin, transferrin, and ferritin, or stored in natural chelators called siderophores (7, 8). For bacteria inside a mammalian host to acquire iron, it is generally extracted from heme, since heme is the most abundant source of iron in mammals.

To date, systems that acquire iron from heme bound to hemoglobin within the host organism have been reported for a variety of pathogens. For example, the Isd (iron-regulated surface determinant) gene cluster encodes a series of cell surface, membrane, and cytoplasmic proteins involved in heme transport in the gram-positive bacteria *Staphylococcus aureus* (9, 10, 11). Subsequent studies revealed that several other gram-positive bacteria such as *Bacillus anthracis* (12, 13) or *Listeria monocytogenes* (14) have a system homologous to Isd, suggesting that this heme acquisition system is widely conserved in many other pathogens. *S*. *pyogenes*, on the other hand, possesses an iron-regulated *sia* operon consisting of 10 genes (15). Among them, *Shr*, *Shp* and *siaABC* (*htsABC*) are the well-studied proteins. SiaABC proteins are ABC-type heme transporters, while Shp is a membrane surface protein that transfers heme to SiaA (16, 17, 18, 19). Shr, the first gene encoded in the *sia* operon, is involved in heme acquisition from hemoglobin and its subsequent transfer to Shp (20, 21) and is known as a fascinating virulent factor (22, 23). For example, loss of the *shr* gene inhibits growth of the MIT1 strain in human peripheral blood and prolongs survival in a mouse model (24). It has also been shown that deletion of the *shr* gene results in prolonged survival in a zebrafish infection model (15). These studies highlight the importance of Shr protein in the host infection process by *S*. *pyogenes*.

At the molecular level, Shr is a protein composed of 1275 amino acid residues comprising several domains (Fig. 1*A*). Previous studies have revealed the role of some of these domains. For example, the two hemoglobin-interacting domains (HID1 and HID2) located at the N-terminal region of Shr capture hemoglobin to extract heme (25); structures of the complex of HID2 with hemoglobin have recently been reported (26, 27). A “cap and release” mechanism that specifically captures heme from the β-chain of hemoglobin has been proposed to explain how heme is extracted from hemoglobin (27). It has also been reported that the pair of NEAT domains bind heme and quickly transfer it to Shp (28) (Fig. 1*B*). Although the knowledge of the function of each domain from Shr is accumulating, a significant part of the structural information is still missing, thus limiting the understanding of how Shr captures and transfers heme to Shp. Atomic details regarding the heme axial ligands in the NEAT domains of Shr are currently unavailable. This is clearly distinctive, considering that many structural reports into how NEAT domains from other gram-positive bacteria recognize heme have been described in detail. Generally, the iron-coordinating residue in those other systems is a tyrosinate (29) and based on that, antimicrobial strategies are being explored (30, 31).

**Figure 1:**
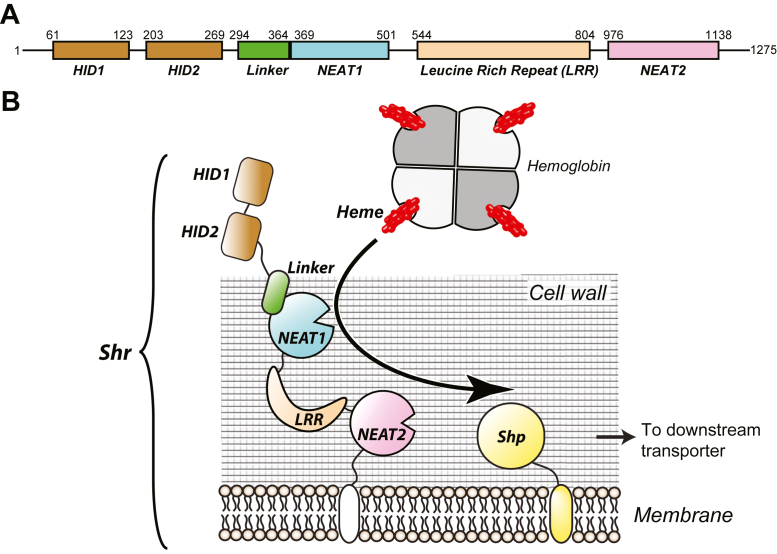
**Heme acquisition by Shr**. *A*, modular structure of Shr. The protein Shr from *S*. *pyogenes* is composed of two HIDs (hemoglobin-interacting domain), a NEAT1 domain with a preceding linker domain, a leucine-rich repeat (LRR) domain, and a NEAT2 domain. *B*, schematic illustration of heme acquisition mediated by Shr. Starting from the interaction of HID2 with hemoglobin, heme is transferred to Shp *via* NEAT domain. However, the detailed mechanism at the molecular level is yet to be explained.

In this study, we have determined the structures of the two NEAT domains within Shr, NEAT1 and NEAT2. We successfully obtained crystal structures of NEAT1 and NEAT2 domains, the former with its preceding linker, at 2.35 Å resolution and 2.66 Å resolution, respectively. Together with mutational analysis, the key role of the coordinating methionine residues in the heme binding pocket was revealed, showing interesting features common to Shp and SiaA, both from *S*. *pyogenes*, but not with the NEAT domains from other gram-positive bacteria. In summary, we have characterized the structural and functional properties of two NEAT domains and discussed the biological relevance of those features.

## Results

### Crystal structure of Linker-NEAT1

**Figure 2:**
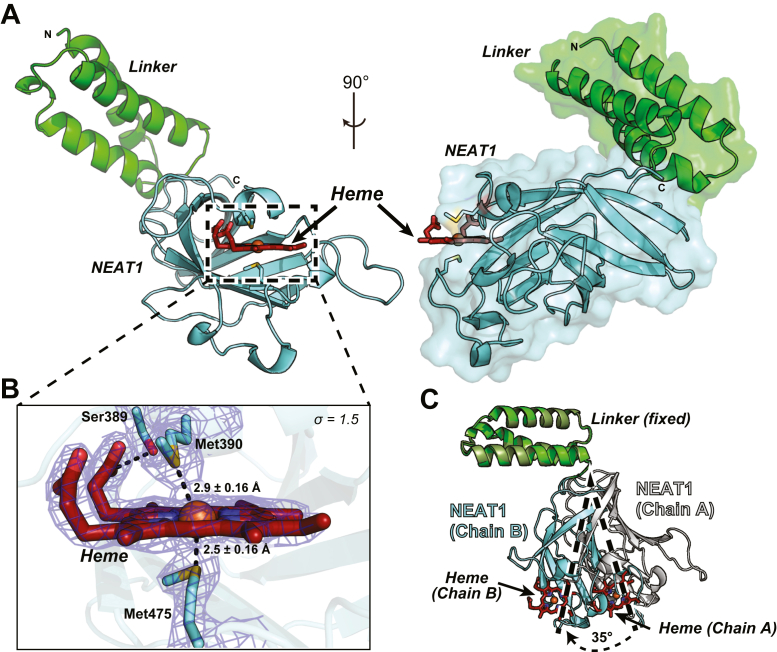
**Structure of Linker-NEAT1 from Shr**. *A*, overall structure from two different angles with surface representation on one of them. The linker region and NEAT1 domain are colored in *green* and *cyan*, respectively. Heme is depicted in firebrick stick, with oxygen and iron atom in *red* and *orange*, respectively. N and C represent the N- or C-terminus of the protein sequence. *B*, close-up view of the heme-binding site in chain B from the asymmetric unit. Heme was found coordinated by two methionine residues, Met390 and Met475 and had a hydrogen bond with Ser389. Oxygen and sulfur atoms are shown in *red* and *yellow*, respectively. The distances between the sulfur atom and the iron atom from the heme are indicated. The distance error was derived from the Cruickshanks DPI (Diffraction Precision Indicator), a parameter that estimates the overall coordinate error in the structure. (56). The distances in chain A are indicated in the main text. The sigma-A weighted 2Fo-Fc electron density map corresponding to the methionine residues and heme is shown contoured at σ = 1.5. *C*, superposition of two Linker-NEAT1 chains from the asymmetric unit of the crystal. Linker region from chain A or B is colored in *drab* or *green*, while NEAT1 domain from chain A or B is colored in *white* or *cyan*. The two chains are aligned by the Linker region. *Dotted lines* and *arrows* show the movement of NEAT1 domain aligned by the linker region.

To determine the structure of NEAT1, we first optimized the expression construct, since it was not possible to purify only the NEAT1 domain due to the visible aggregation occurring during the purification process, an observation already reported in the literature (32). We prepared a construct termed Linker-NEAT1 comprising amino acid residues Ser294 to Gly502 that included the linker region (25, 33). This construct was successfully expressed and employed for the preparation of protein crystals. The crystal structure of Linker-NEAT1 was subsequently determined at 2.35 Å resolution (Fig. 2*A*, Table 1). The visible residues in the electron density comprised Glu298 to Lys501 in chain A and Leu297 to Lys501 in chain B (we could locate two independent protein chains in the asymmetric unit), and a single heme moiety bound to each protein chain. The linker region was composed of a three α-helix motif, whereas the NEAT1 domain predominantly consisted of β-sheet secondary structure elements. A characteristic feature of the NEAT1 domain of Shr was the presence of two methionine residues, Met390 and Met475, that are employed by the protein to coordinate heme (Fig. 2*B*). The distance between the sulfur atom of Met390 in Chain A of the asymmetric unit and the iron atom of the bound heme in the same chain was 3.1 ± 0.16 Å, whereas in Chain B of the asymmetric unit, that distance was 2.9 ± 0.16 Å. For the other axial ligand, Met475, the distances between the sulfur atom and the iron atom of heme in chain A and in chain B were 2.6 ± 0.16 Å and 2.5 ± 0.16 Å, respectively. These data indicate that the distance between Met475 to the iron atom was slightly shorter than that observed between Met390 and the same iron atom. We thus hypothesized that Met475 could play a more prominent role in heme binding than Met390, although that hypothesis would need to be further evaluated (see below). Other than these two methionine residues, Ser389 formed a hydrogen bond with the tetrapyrrole's propionate at position seven in ring D (Table S1). In addition to these coordinating interactions, many hydrophobic residues seem to contribute to the formation of the heme-binding pocket. As a result, the interface area, an average value of the buried surface area (BSA) resulting from the binding of heme to NEAT1, was calculated to be 440 Å^2^ by the PISA server (34).

**Table 1:** Data collection and refinement statistics^a^

Data collection	Linker-NEAT1	NEAT2
Space group	P 1 2_1_ 1	I 2 2 2
Unit cell		
a, b, c (Å)	42.58, 113.25, 44.28	67.68, 99.05, 123.76
α, β, γ (°)	90.0, 96.4, 90.0	90.0, 90.0, 90.0
Resolution (Å)	42.32–2.35	41.47–2.66
Wavelength	1.007	1.0000
Reflections (all)	123,791 (11,951)	155,441 (22,077)
Reflections (unique)	17,354 (1669)	12,321 (1621)
*R*_merge_	0.137 (0.697)	0.063 (1.001)
*R*_p.i.m._	0.055 (0.277)	0.019 (0.279)
CC_1/2_	0.996 (0.865)	1.000 (0.898)
*I/σ(I)*	11.0 (2.8)	23.3 (2.6)
Multiplicity	7.1 (7.2)	12.6 (13.6)
Completeness (%)	99.8 (99.3)	100.0 (100.0)
Refinement statistics		
Resolution (Å)	42.32–2.35	41.47–2.66
*R*_work_/*R*_free_ (%)	22.0/26.8	22.0/27.6
No. Protein chains	2	2
No. atoms		
Protein	3190	2438
Heme	86	86
Water	71	9
Others	14	35
B-factor (Å^2^)		
Protein	39.14	87.27
Heme	38.89	84.73
Water	28.42	73.622
Others	33.37	123.137
Ramachandran plot		
Preferred (%)	92.9	89.6
Allowed (%)	7.2	10.4
Outliers (%)	0.0	0.0
RMSD bond (Å)	0.0069	0.074
RMSD angle (Å)	1.5	1.6
PDB	9W5Z	9W5Y

a Statistics values given in parenthesis refer to the highest resolution bin.

When the two protein chains found in the asymmetric unit were superimposed, a visible difference in the angle of the linker region with respect to the NEAT1 domain was observed (Fig. 2*C*). The difference in the angle between the linker region and NEAT1 in each copy was 35°, indicating the flexible nature of NEAT1 domain with respect to the linker region. Indeed, there was only a few hydrogen bonds formed between the linker region and the NEAT1 domain (Figs. S1, *A* and *B*, Table S2), supporting the idea of a flexible hinge between the two domains. These characteristics are different with respect to the linker next to the NEAT3 domain of the IsdH protein from *S*. *aureus* (35). The linker region of IsdH Linker-NEAT3 also forms a three α-helix motif, but the length and topology of the helix motif, and the relative position of the linker region with respect to the NEAT domain, are different with respect to the linker in Shr (Fig. S1*C*). Given the fact that the NEAT1 domain of Shr cannot be purified without the linker region, and that the linker region does not seem to strongly interact with the NEAT1 domain, the linker region may improve the colloidal stability of NEAT1 and/or facilitate the correct folding of NEAT1.

### Characterization of Linker-NEAT1

**Figure 3:**
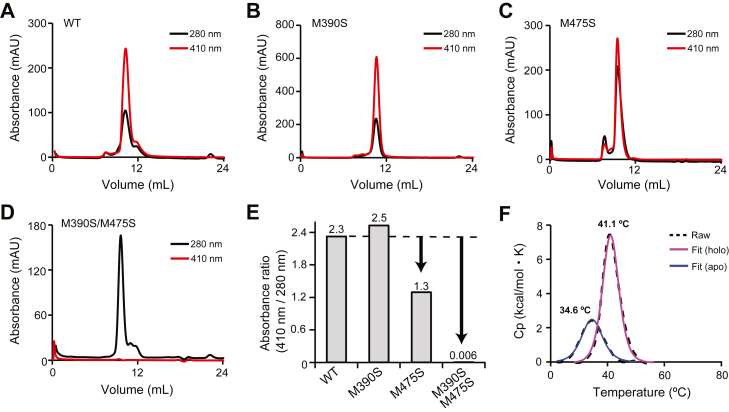
**Characterization of Linker-NEAT1**. Size exclusion chromatography of Linker-NEAT1 mutants in complex with heme: (*A*) WT, (*B*) M390S, (*C*) M475S, and (*D*) M390S/M475S. *Black* or *red solid lines* corresponded to the absorbance at 280 nm and at the Soret band (410 nm), respectively. *E*, comparison of the absorbance ratio at the Soret band with respect to 280 nm. The ratio values from each mutant are shown at the top of the bar graph. *F*, thermostability analysis by DSC. *Dotted lines* show the raw data, and solid lines are fitted data. Profiles from heme-bound (holo) form or heme-unbound (apo) form are shown in *blue* or *pink lines*, respectively. See also Figure S2 for the full raw data.

To evaluate the contribution of the methionine residues identified above (Met390 and Met475) to the binding of heme, we proceed to carry out mutational analysis of Linker-NEAT1. Here, we employed an assay using size exclusion chromatography (SEC) to monitor the binding ability of the methionine mutants to heme by observing how they co-elute (36). Because some of the methionine to alanine mutants in this study were insoluble (we could not purify them), we instead prepared methionine to serine mutants, resulting in the preparation of M390S, M475S, and the double mutant M390S/M475S, of Linker-NEAT1. The heme-free protein (also termed unbound or apo) was mixed with heme to prepare heme-bound (holo) form. The protein samples were then subjected to SEC, and the absorbance at 280 nm and 410 nm was monitored to determine if the protein co-eluted with heme. As shown in Figure 3, *A*–*D*, a distinct absorbance peak at 410 nm appeared in all Linker-NEAT1 samples except for that of the double mutant M390S/M475S, indicating that the heme is bound to Linker-NEAT1 WT, M390S and M475S mutants, but not to the double mutant. The absorbance ratio at 280 nm and 410 nm was highest in Linker-NEAT1 WT, showing 2.3 times higher absorbance at 410 nm that at 280 nm. A similar trend was observed in M390S mutant, although the absorbance at 410 nm of the M475S mutant was only 1.3 times greater than that at 280 nm, indicating that a lower fraction of proteins retained heme. This could be interpreted as that the contribution of Met475 for binding was indeed more significant than that of Met390, something that was hypothesized from the sulfur-iron distances determined from the crystallographic data (Figs. 2*B*, 3, *B*–*C*). When both methionine residues were substituted to serine, the heme-binding activity was completely abrogated (Fig. 3*D*). The ratio of absorbance at 280 nm and 410 nm is summarized in Figure 3*E*.

We subsequently evaluated the thermostability of Linker-NEAT1 using differential scanning calorimetry (DSC). The transition midpoint (also known as melting temperature or *T*m) of Linker-NEAT1 in the absence of heme was 34.6 °C. In the presence of heme, the *T*m increased to 41.1 °C (Fig. 3*F*, Fig. S2*A*). Although the binding of heme slightly stabilized the overall protein folding, this data explains why NEAT1 domain from Shr is unstable compared with, for example, IsdH Linker-NEAT3 (*T*m = 73.2 °C in the presence of heme) (35). In the raw data from DSC, there was a small peak around 70 °C (Fig. S2*A*) of unknown nature. To cross-validate the DSC data, we employed differential scanning fluorometry (DSF), which confirmed that the transition around 30 to 40 °C corresponded to the main melting transition of the polypeptide chain (Fig. S2*B*). The DSF data excludes the possibility that the peaks observed by DSC at around 70 °C are the consequence of protein unfolding (considered as the loss of tertiary structure driven by the exposure of the protein hydrophobic core), although we cannot completely preclude that these small peaks reflect partial unfolding of particular secondary structure elements.

It is also observed that the values of *T*_m_ determined by DSF are lower than those determined by DSC, which could reflect the detection of the early stages of exposure of the hydrophobic region in the DSF experiment, before the main cooperative unfolding event absorbing the maximum amount of heat, is detected by DSC. The values of *T*_m_ of Linker-NEAT1 or NEAT2 did not significantly change in the double mutant with respect to WT, suggesting that individual mutations of the axial ligands also will not affect the overall stability of the proteins (Fig. S2*C*). Collectively, both DSC and DSF have indicated that Linker-NEAT1 displays an unusually low thermal stability, whereas NEAT2 possesses a notably greater stability that further increases in the presence of heme.

### Crystal structure of NEAT2

**Figure 4:**
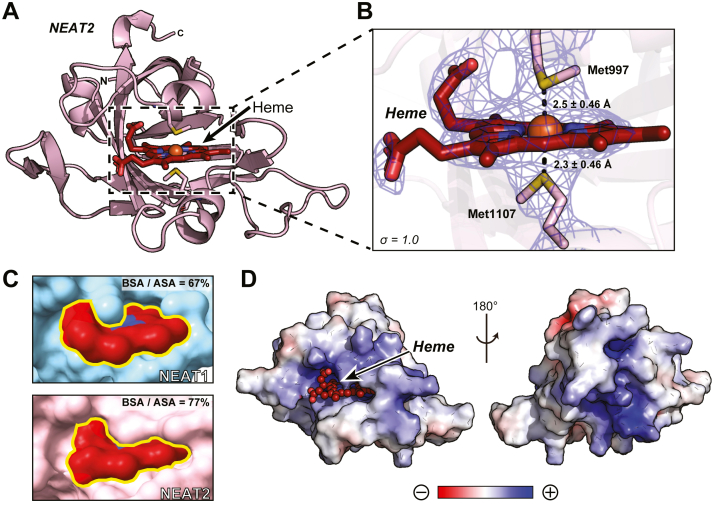
**Crystal structure of NEAT2 from Shr**. *A*, whole structure of NEAT2. Heme is represented with firebrick sticks. Oxygen, nitrogen, sulfur and iron atoms are depicted in *red, blue, yellow* and *orange*, respectively. N and C indicate the N- or C-terminus of the protein sequence. *B*, Close-up view of the heme-binding site in chain *B*. Heme was found coordinated by two methionine residues, Met997 and Met1107. The distances between sulfur atom and the iron atom from heme are shown, including the distance error calculated from the Cruickshanks DPI (56). The distances in chain A are indicated in the main text. The sigma-A weighted 2Fo-Fc electron density map corresponding to the methionine residues and heme is shown at the contouring level of σ = 1.0. (*C*) Surface representation of heme molecule bound to the pocket of NEAT1 (*cyan*) or NEAT2 (*pink*). The BSA/ASA ratio is calculated based on PDB PISA (34) analysis. Larger values indicate a greater portion of the heme molecule buried in the binding pocket. *D*, electrostatic potential of NEAT2 surface. The color gradient from *red* to *blue* represents negative (−5 kT) to positive electrostatic potentials (+5 kT).

We also aimed at obtaining the crystal structure of the second NEAT domain in Shr, termed NEAT2. The construct comprised residues Ala970 to Thr1129 and was successfully purified and crystallized. The crystal structure of NEAT2 was determined at 2.66 Å resolution (Fig. 4*A*, Table 1). The RMSD between NEAT1 and NEAT2 was calculated to be 1.7 Å, and the predominant secondary structure in NEAT2 was also β-sheet, showing a substantial degree of structural similarity between the two NEAT domains. The asymmetric unit contained two copies of NEAT2 in complex with heme. Analogously to Linker-NEAT1, NEAT2 also employed two methionine residues (Met997 and Met1107) to coordinate heme. The distances between the iron atom and the sulfur atom of the axial ligands Met997 and Met 1107 were 2.9 ± 0.46 Å and 2.7 ± 0.46 Å in chain A, and 2.5 ± 0.46 Å and 2.3 ± 0.46 Å in chain B. The difference in the Met-iron distances between the two axial ligands and iron in NEAT2 is smaller than in NEAT1, suggesting a more balanced contribution of each Met to heme binding in NEAT2 with respect to that in NEAT1 (Fig. 4*B*). Another difference is related to the architecture of the heme binding pocket: NEAT2 possesses a longer and more extended loop surrounding heme resulting in the formation of a deeper heme-binding pocket (Fig. S3). First, the volumes of the heme-binding pocket in NEAT2 and Linker-NEAT1 calculated by PyVOL (37) were 490 Å^3^ and 373 Å^3^, respectively. Second, and related to the previous consideration, the heme moiety bound to NEAT2 is buried deeper in the pocket compared with that in NEAT1, as revealed by the ratio of BSA over the accessible surface area (ASA, defined as the solvent-accessible area of a residue or a ligand). The BSA/ASA ratio for heme bound to NEAT1 was determined to be 67%, whereas the same ratio for heme bound to NEAT2 was 77% (Fig. 4*C*, Table S3). Consistent with this observation, the interface area between NEAT2 and heme determined by the from PISA server (34) was 502 Å^2^, approximately 61 Å^2^ larger than that in NEAT1. Another characteristic feature of NEAT2 was its high pI value (9.62). A simple calculation of its electrostatic potential was performed using the default parameters in the APBS software package in PyMOL (38) showing a large positively charged surface around and even beyond the heme-binding region (Fig. 4*D*). It is likely that under neutral pH conditions, the positive charge of NEAT2 facilitates the association of the protein with the negatively charged cell membrane.

### Characterization of NEAT2

**Figure 5:**
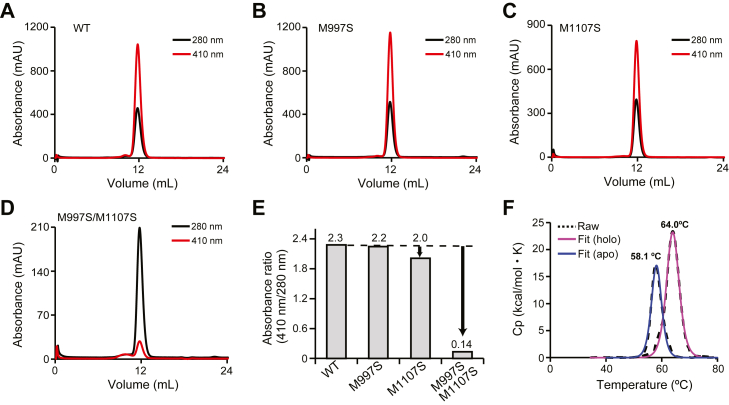
**Characterization of NEAT2**. Size exclusion chromatography of NEAT2 in complex with heme: (*A*) WT, (*B*) M997S, (*C*) M1107S, and (*D*) M997S/M1107S. *Black* and *red solid lines* correspond to absorbance at 280 nm and the Soret band at 410 nm, respectively. *E*, comparison of the absorbance ratio at 410 nm with respect to 280 nm. The values from each mutant are shown at the top of the bar graph. Unlike Linker-NEAT1, only the double mutant showed a clear reduction of heme-binding. *F*, thermostability analysis by DSC. *Dotted lines* and solid lines correspond to the raw data and the fitted data, respectively. Chromatographic profiles from heme-bound (holo) form or heme-unbound (apo) form are shown in *magenta* and *blue solid lines*, respectively. See also Figure S2 for the full raw data.

To examine the precise role of each Met residue, we performed mutation analysis in NEAT2. First, the contribution of each methionine residue to binding of heme was monitored by SEC. Among the mutants tested (NEAT2 WT, M997S, M1107S and M997S/M1107S), only M997S/M1107S showed a drastically lower heme-binding ability. The single mutation of methionine did not have a significant influence, and the degree of heme binding in the mutants was comparable to that of the WT protein (Fig. 5, *A*–*E*). These results suggest that, in NEAT2, the presence of a single axial methionine is sufficient to retain heme in the binding pocket. This observation is consistent with the distance between sulfur and iron atom in the crystal structure, again showing a close correlation between mutation analysis and structural data.

Thermostability of NEAT2 was also assessed by DSC analysis. The melting temperature of NEAT2 in the absence and presence of heme was determined to be 58.1 °C and 64.0 °C, respectively (Fig. 5*F*, Fig. S2, *A* and *B*). The data clearly indicate that the tertiary structure of NEAT2 is significantly more stable than Linker-NEAT1. The value of *T*m of NEAT2 exceeded more than 20 degrees that of NEAT1. In addition, the deeper heme-binding pocket of NEAT2 results in greater stabilization when heme binds to NEAT2 than when binding to NEAT1, suggesting that NEAT2 interacts with heme with higher affinity (see below).

### Heme transfer experiments between NEAT domains and Shp

To qualitatively evaluate the heme transfer activity of Linker-NEAT1 and NEAT2, we performed heme transfer assays. In a previous study, it was shown that heme could be transferred not only from NEAT1 to the heme-binding domain of Shp, but also from NEAT2 to NEAT1 (28), which is somehow controversial given that the affinity of heme for NEAT2 was greater than that for NEAT1. Herein, we have revisited the heme transfer activity among Linker-NEAT1, NEAT2 and Shp. In this experiment, the heme-loaded form of the donor protein containing a hexa-histidine tag was mixed with the heme-free form of the acceptor protein. After mixing donor and acceptor, the proteins were separated using a Ni-NTA agarose resin by collecting the acceptor protein from the flowthrough and the donor protein from the eluted fraction (Fig. S4). The absorbance spectrum of the proteins was examined before and after the heme transfer reaction to qualitatively monitor the degree of heme transfer between donor and acceptor.

**Figure 6:**
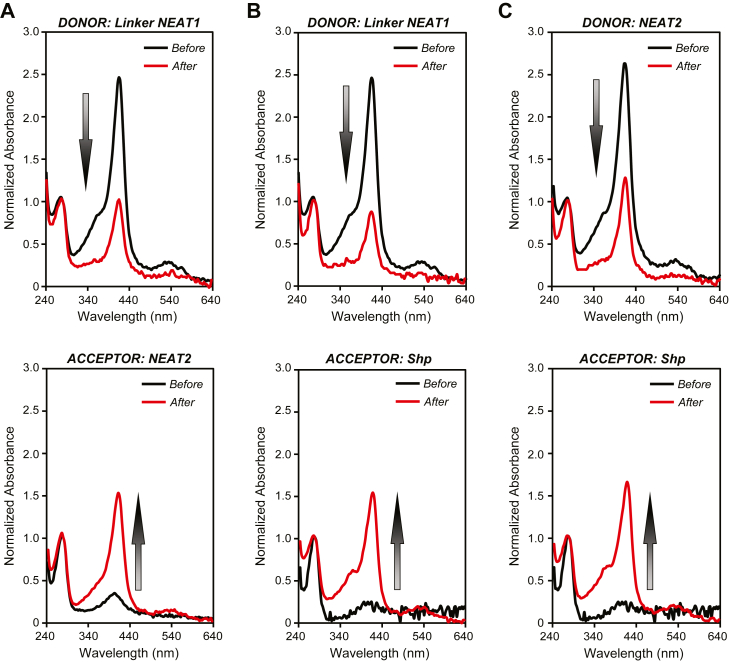
**Heme transfer assay**. *A*, heme transfer experiment from Linker-NEAT1 to NEAT2, (*B*) from Linker-NEAT1 to Shp, and (*C*) from NEAT2 to Shp. In each panel, the upper and lower spectra show those from donor or acceptor of heme. *Black* and *red lines* correspond to the absorption spectra before and after the transfer reaction, respectively. In all experiments, the absorbance at the Soret band decreased in the donor proteins, while the value increased in the acceptor proteins. The arrows emphasized the change of absorbance at the Soret band before and after the transfer reaction.

We first examined three pairs of donor/acceptor proteins, specifically Linker-NEAT1/NEAT2, Linker-NEAT1/Shp and NEAT2/Shp. In these experiments, the donor proteins were prepared by loading hemin chloride (ferric form of heme) to the proteins purified in the heme-free form. In all transfer reactions, the absorbance of the Soret band of the donor protein significantly decreased after the heme transfer reaction, which was correlated with a concomitant increase of absorbance of the Soret band of the acceptor protein (Fig. 6, *A*–*C*). As a control, we verified by SDS/PAGE that the separation of donor from acceptor was effectively achieved (Fig. S5). Collectively, these results indicate that heme transfer occurs from Linker-NEAT1 to NEAT2, from Linker-NEAT1 to Shp, and from NEAT2 to Shp.

We further examined whether the heme transfer occurs in the reverse directions, *i*.*e*. from NEAT2 to Linker-NEAT1, from Shp to Linker-NEAT1, and from Shp to NEAT2. In the experiment, only small changes in the absorbance of the donor and acceptor proteins at the Soret band were observed before and after the transfer reaction took place (Fig. S6, *A*–*C*), indicating a meager transfer of heme from donors to acceptors in the reverse transfer experiments.

We note that in the reverse transfer experiments, the donor proteins were acquired from the expression host in the heme-bound form, *i*.*e*. the receptors containing the endogenous heme produced in *Escherichia coli*, and indeed the position of the Soret band of NEAT2 and Shp before the transfer reaction appeared at greater values of wavelength (∼425 nm) (Table S4). The position of the Soret band is determined by several factors, among them the oxidation state of the central iron atom (32). The wavelength observed in the donor suggested some preference for the ferrous form to bind the receptor, although we also note that at the end of the transfer reaction, all heme receptors showed a clear predominance of the ferric form (∼415 nm). To make a direct comparison with the data in Figure 6, we also examined the heme transfer of NEAT2 bound to ferric heme. To that end, we added hemin chloride (ferric heme) to the protein purified as the heme-free form. As shown in Figure S6*D*, the amount of transferred heme from NEAT2 to linker-NEAT1 was noticeably smaller than that observed in the forward direction (Fig. 6), suggesting similar trends in the other donor-acceptor pairs. Collectively, these results indicate that heme transfer occurs in one direction to maximize efficacy.

To rationalize the transfer data above, we determined the affinity of heme for each of the three heme-receptors by isothermal titration calorimetry (ITC). It is observed that the affinity increases in the same order as that of the heme-transfer above, *i*.*e*., heme binds with increasing strength in the sequence Linker-NEAT1 < NEAT2 < Shp (Fig. S7 and Table S5). The correlation between heme transfer and heme binding suggests that the movement of heme is thermodynamically controlled (equilibrium). Another interesting aspect revealed by the ITC data is that binding of heme to the NEAT domains and to Shp is governed by the change of entropy, with only a small contribution from the change of enthalpy. This observation stands in stark contrast with the heme-binding NEAT domain from, for example, IsdH from *S*. *aureus*. This difference may be related to the nature of the axial ligands of iron - two methionine residues in the NEAT domains of Shr and in Shp, but a single tyrosine in the NEAT3 domain of IsdH (see below in the Discussion section). Collectively, the results in this section indicate that heme transfer occurs in one direction to maximize the transfer efficiency, and it is driven by the increasing affinity of the heme receptors.

## Discussion

In this study, we successfully determined the crystal structure of Linker-NEAT1 and NEAT2 of Shr protein from *S*. *pyogenes*. So far, structures of 10 different NEAT domains including those from this study from gram-positive bacteria have been reported (Table S6), namely IsdA NEAT domain (PDB ID: 2ITF) (39), IsdB NEAT2 (PDB ID: 3RTL) (40), IsdC NEAT domain (PDB ID: 2O6P) (41), IsdH NEAT3 (PDB ID: 2Z6F) (42) from *S*. *aureus*, IsdX1 NEAT domain (PDB ID: 3SIK) (13) and IsdX2 NEAT5 (PDB ID: 4H8P) (12) from *B*. *anthracis*, Hbp2 NEAT2 (PDB ID: 4MYP) (14) from *L*. *monocytogenes* and Shp (PDB ID: 2Q7A) (43), Shr NEAT1 (determined in this study, PDB ID:9W5Z) and Shr NEAT2 (determined in this study, PDB ID:9W5Y) from *S*. *pyogenes*. When the reported NEAT domains were compared with Linker-NEAT1 or NEAT2 of Shr, there was a trend in the RMSD value; NEAT domains from *L*. *monocytogenes* or *B*. *anthracis* showed smaller RMSD values (ranging 1.4 Å to 1.7 Å) than those from *S*. *aureus* (ranging 1.6 Å to 2.3 Å), while Shp showed the greatest value among them (2.7 Å to Linker-NEAT1 or 2.4 Å to NEAT2).

**Figure 7:**
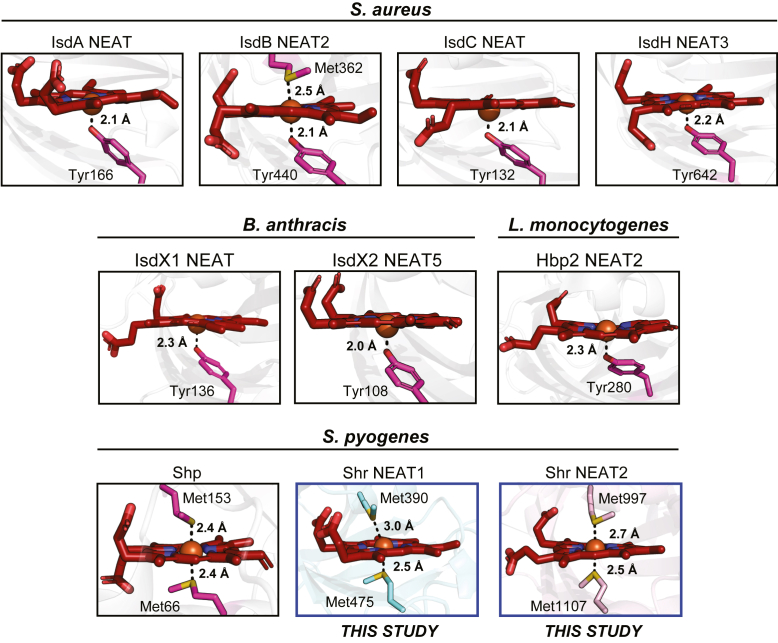
**Comparison of NEAT domains from other gram-positive bacteria**. Heme-binding sites from all heme-binding NEAT domains that are currently available in the PDB are shown. The structures shown are following; IsdA NEAT (PDB ID: 2ITF), IsdB NEAT2 (PDB ID: 3RTL), IsdC NEAT (PDB ID: 2O6P) and IsdH NEAT3 (PDB ID: 2Z6F) from *Staphylococcus aureus*, IsdX1 NEAT (PDB ID: 3SIK) and IsdX2 NEAT5 (PDB ID: 4H8P) from *Bacillus anthracis*, Hbp2 NEAT2 (PDB ID: 4MYP) from *Listeria monocytogenes* and Shp (PDB ID: 2Q7A), Shr NEAT1 and Shr NEAT2 from *Streptococcus pyogenes*. All NEAT domains except the ones from *S*. *pyogenes* utilized Tyr as a coordinating residue, while methionine residues are preferentially employed in *S*. *pyogenes*. Residues used to bind to heme are depicted in stick and colored in magenta. The distances between the oxygen atom of the tyrosine residue and the iron atom from heme or the sulfur atom of methionine residues and the iron atom from heme are shown.

On the one hand, these values indicate that there is a significant degree of structural homology among the NEAT domains from various species. On the other hand, the residues employed to coordinate heme in *S*. *pyogenes* are different to those of other species. In *S*. *pyogenes*, all NEAT domains reported so far, and that includes the two NEAT domains reported in this study, employ methionine to coordinate heme, whereas the NEAT domains from *S*. *aureus*, *B*. *anthracis* or *L*. *monocytogenes* use tyrosinate (Fig. 7). Tyrosinate is an anionic axial ligand, stabilizing Fe(III)-porphyrin (so-called ferric form of heme) to adjust the charge interaction between heme moiety and axial ligand as shown by previous studies (29). In contrast, since methionine is a neutral ligand, this residue may coordinate both Fe(II)- or Fe(III)-porphyrin. This characteristic would allow *S*. *pyogenes* to adapt to various conditions in the host environment. For example, while the ferrous form of heme may be more abundant in oxygen-rich environment in blood circulation, ferric form of heme is also quickly produced in inflammatory oxidative lesions (44). By having the ability to bind to both forms of heme, Shr ensures that *S*. *pyogenes* will consistently capture heme regardless of the oxidative state of the iron atom.

**Figure 8:**
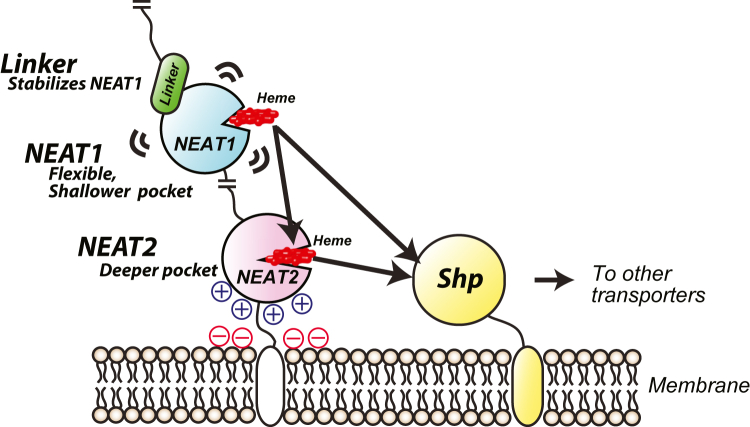
**Model of heme transfer in Shr from *S*. *pyogenes***. The *black arrows* represent possible pathways for heme transfer consistent with our data. One possibility is that heme transfer starts from NEAT1 and heme is transfer to Shp (direct transfer). Another possibility is that heme is transfer to Shp *via* NEAT2. This study revealed the role and characteristics of each domain. The linker region of NEAT1 is necessary to stabilize the NEAT1 domain. NEAT1 is a flexible domain with a shallow heme-binding pocket, allowing the active transfer of heme from this domain either to NEAT2 or Shp. On the other hand, NEAT2 has a deeper heme-binding pocket. Also, the positive charge of the surface of NEAT2 can allow this domain to be resist in the proximity of the membrane, which has a negative charge. These characteristics are consistent with the idea that this domain is used mainly to store heme on the membrane surface and release it to Shp depending on the bacterial needs.

The structural information and characterizations performed in this study also give insights into how heme is transferred from the Shr protein to Shp (Fig. 8). DSC data revealed that Linker-NEAT1 displays low thermostability (Fig. 3*F*), presumably reflecting the flexible and dynamic behavior of this domain. Also, the pocket formed by NEAT1 was shallower than that of NEAT2 and thus showed smaller value of BSA/ASA ratio compared to NEAT2 (Fig. 4*C*). These results suggest that Linker-NEAT1 is actively employed to catch and quickly release heme to the acceptor domain such as NEAT2 or Shp protein. Similarly, the presumably weaker interaction between linker region and NEAT1 (Fig. S1) would preferably work for the NEAT1 domain to actively move around and catch heme by increasing the probability of NEAT1 domain to encounter the acceptor proteins. In the IsdH protein, the linker region and NEAT3 has intact intramolecular interaction, and both linker region and NEAT3 domain facilitates the conformational change in the hemoglobin upon binding to it (35). However, considering the clear difference in the intramolecular interaction and topology of the linker region and NEAT domain in both proteins (Fig. S1), the role of linker region in IsdH does not necessarily apply to Shr Linker-NEAT1. The NEAT2 domain, on the contrary to Linker-NEAT1 domain, displayed greater thermostability (Fig. 5*F*) by more than 20 °C, and deeper heme-binding pocket compared with Linker-NEAT1 (Fig. 4*C*), consistent with stronger affinity for the heme moiety and thus less active in transferring heme to Shp (Figs. 6, S7, Table S5). Indeed, previous studies pointed out that NEAT2 is used as a heme-storage domain (27), an idea well aligned to our analysis herein. Given these observations, the main heme transfer route from Shr to Shp could be that from Linker-NEAT1 to Shp, while a pathway *via* NEAT2 could be employed as a secondary pathway that would be ready to be activated when necessary. Having an additional pathway would enable both quick and sustainable supply of heme into the bacterial cells, increasing the likelihood that *S*. *pyogene* survives in various environments within the host tissue.

Collectively, in this study, we have characterized the two NEAT domains from the Shr protein of *S*. *pyogenes* through structural and biophysical analysis. Although the role of methionine residues in the heme-binding activity was similar in the two NEAT domains, it also displayed some distinctive differences. Collectively, these characteristics facilitate versatile heme transfer mechanisms in *S*. *pyogenes*. Also, the comparison of NEAT domains among species provides important insights into the survival strategies adopted by pathogenic bacteria.

## Experimental procedures

### Cloning of NEAT constructs

A gene coding Shr full-length was amplified from the whole genome of *S*. *pyogenes* ssi-1 strain by KOD-one PCR master kit (Toyobo) following the manufacture’s instruction and cloned into the expression vector pET28b by the Hifi DNA assembly cloning method (New England Biolabs). From this expression vector, Linker-NEAT1 (Ser294 to Gly502), NEAT2 (Ala970 to Thr1129) and Shp (Asp30 to Thr180) was subcloned into another pET28b vector. All constructs contained an N-terminal His_6_-tag followed by TEV protease cleavage site, which was subsequently used to remove the tag. For the site-directed mutagenesis, KOD-plus mutagenesis kit (Toyobo) was used following the manufacturer’s instruction.

### Expression and purification of recombinant proteins

*E*. *coli* BL21(DE3) competent cells (NIPPON GENE) were transformed with the expression vector described above. Expression of heme-unbound form of each construct was conducted in M9 minimal medium following the previous study (45), while that of heme-bound form of each construct was conducted in LB medium. Both media were supplemented with 50 ng/μl kanamycin. In both media, bacteria were grown at 37 °C and induction of the recombinant protein by the addition of 0.5 mM isopropyl-β-D-thiogalactopyranoside (IPTG) (BIOSYNTH) was carried out when the O.D._600_ value reached 0.5. After the induction, the culture temperature was set at 20 °C and cells were collected in 16 h.

Cells were harvested by centrifugation at 4 °C, 10 min, 7000×*g* and then resuspended in binding buffer (20 mM Tris, 500 mM NaCl, 20 mM Imidazole pH 8.0). The resuspended cells were sonicated for 5 min on ice. The soluble fraction was collected by centrifugation at 4 °C, 60 min, 23,500×*g*. The supernatant was filtered with a 0.8-μm sterile filter and loaded into a gravity column containing 1 ml of Ni-NTA agarose (Fujifilm Wako) to do immobilized-metal affinity chromatography (IMAC). The resin was equilibrated with the binding buffer in advance. After washing the column with 10 CV of the binding buffer, the protein of interest was eluted with the elution buffer (20 mM Tris, 500 mM NaCl, 300 mM Imidazole pH 8.0) at 5 CV. To remove the His_6_tag and TEV cleavage sequences, TEV protease at a mass ratio of 1:10 (TEV protease: protein of interest) was added and simultaneously dialyze against the SEC buffer (20 mM Tris, 500 mM NaCl pH 8.0) at 4 °C for 16 h. The cleaved tag, TEV protease also containing His_6_tag (prepared in the following method) and protein of interest without His_6_tag was separated by loading the sample into Ni-NTA agarose resin again and collecting the flowthrough. The resultant protein was further purified in size exclusion chromatography (SEC) using a HiLoad 16/600 superdex75 pg (Cytiva) equilibrated with the SEC buffer. The SEC experiments to purify the proteins were performed using an AKTA Go instrument (Cytiva) or AKTA FPLC instrument (GE Healthcare). The target proteins were collected and concentrated at around 1 mg/ml using AMICON 10 kDa cut-off and immediately frozen under liquid nitrogen and kept at −80 °C until the experiments. In case of crystallization of Linker-NEAT1 or NEAT2, a french press at 1 kbar was used to lyse the bacterial cells instead of sonication. Also, in case of heme transfer experiments, donor protein had a His_6_-tag. Therefore, we omit the addition of TEV protease and the second IMAC from the methods above.

For the expression of TEV protease, the gene coding the TEV protease with a His_6_tag at C-terminus that was cloned in an expression vector pET28b was expressed in *E*. *coli* BL21(DE3) strain. Following the same protocol stated above, the elution fraction from the IMAC was collected and dialyzed against the buffer (20 mM Tris, 300 mM NaCl, 10% glycerol, pH 7.5). The protein was concentrated at 2 mg/ml and frozen quickly in liquid nitrogen in the storage buffer (20 mM Tris, 300 mM NaCl, 50% glycerol, pH 7.5).

### Crystallization of Linker-NEAT1 and NEAT2

To completely saturate the heme binding to the NEAT domain, frozen heme-bound form of Linker-NEAT1 or NEAT2 protein samples were thawed and mixed with hemin chloride (Tokyo Chemical Industry) dissolved in DMSO (Fujifilm Wako) at a molar ratio protein:heme of 1:3. After incubation with the hemin at room temperature for 5 min, the excess amount of heme was removed by loading the sample to the PD-10 desalting column (Cytiva) equilibrated with crystallization buffer (20 mM Tris, 50 mM NaCl pH 8.0). The protein was further purified with size exclusion chromatography to remove the partially aggregated protein or weakly bound heme from the monomeric protein using a 10/300 superdex75 increase (Cytiva) equilibrated with the crystallization buffer (20 mM Tris, 50 mM NaCl pH 8.0). The peak fraction containing Linker-NEAT1 or NEAT2 was concentrated up to 6.5 mg/ml or 12.5 mg/ml, respectively. Crystals of Linker-NEAT1 or NEAT2 were grown in 0.1 M BIS-TRIS pH6.5, 25% w/v Polyethylene glycol 3350 or 0.2 M Ammonium sulfate, 0.1 M Sodium acetate trihydrate pH 4.6, 30% w/v Polyethylene glycol monomethyl ether 2000, respectively, in a sitting drop method. Suitable crystals were harvested after being briefly incubated with mother liquor supplemented with 20% glycerol and transferred to liquid nitrogen for storage until data collection.

### Data collection and refinement

Diffraction data from a single crystal of Linker-NEAT1 or NEAT2 were collected in beamline BL1A or BL5A at the Photon Factory (Tsukuba), respectively, under cryogenic conditions (100K). Diffraction images were processed with the program XDS (46) and merged and scaled with the program Aimless of the CCP4suite (47). The structure of the protein was determined by the molecular replacement method using the coordinates predicted by AlphaFold2 or AlphaFold3 (48, 49) with the program PHASER (50). The model was refined with the programs REFMAC5 (51) and built manually with COOT (52). Validation was carried out with PDBsum (53). Data collection and structure refinement statistics are given in Table 1. Crystallographic figures were prepared using PyMOL (Schrodinger, LLC.) or UCSF Chimera X (54).

### Heme binding assay of mutants using size exclusion chromatography

The role of each methionine residues in binding with heme was investigated using size exclusion chromatography following the previous study with some modifications (36). Briefly, 25 μM of Linker-NEAT1 or NEAT2 (WT or mutants) prepared in heme-unbound form in the SEC buffer (20 mM Tris, 500 mM NaCl, pH 8.0) was mixed with equimolar of hemin chloride dissolved in SEC buffer with 5% DMSO for a minute. After the incubation, the weakly bound heme was removed by PD-10 desalting column. The collected protein samples were applied to size exclusion chromatography using 10/300 superdex75 increase (Cytiva) and absorbance at 280 nm and 410 nm were monitored simultaneously in an AKTA explorer instrument (GE Healthcare) equipped with multiwavelength monitor UV-900.

### Differential scanning calorimetry

Thermostability of each NEAT domain was measured by differential scanning calorimetry (DSC) in a PEAQ-DSC instrument (Malvern). Proteins were dialyzed into PBS buffer, and the concentration was adjusted to 1 mg/ml. Measurements were performed at a scan rate of 1 °C per minute from 10 °C to 90 °C. Filtered PBS buffer from the dialysis was used as the reference sample to obtain the baseline. The melting temperature *T*m was determined using MicroCal PEAQ-DSC software in the instrument.

### Differential scanning fluorimetry

To cross-validate the DSC results, DSF was performed using a CFX Connect Real-Time System (Bio-Rad). Each sample of Linker-NEAT1 or NEAT2 at 0.2 mg/ml in PBS buffer was mixed with SYPRO Orange Protein Gel Stain (500,00x concentrated in dimethyl sulfoxide (DMSO); Thermo Fisher Scientific) in a final concentration of 500x SYPRO Orange. The protein solution was loaded onto Hard-Shell 96-well PCR Plates (Bio-Rad). The denaturation of each protein was evaluated by measuring fluorescence emitted from SYPRO Orange upon binding to the exposed hydrophobic surface. The melting temperature of the protein (*T*m) was calculated based on the derivatives of relative fluorescent unit (RFU) over the derivatives of temperature (-d(RFU)/dT).

### Isothermal titration calorimetry

To determine the binding affinity of heme to each NEAT domain, we employed isothermal titration calorimetry (ITC) in an AUTO-iTC200 instrument (Malvern). The cell contained the protein sample at 20 μM and the syringe was loaded with a heme solution at 300 μM. The buffer of the samples was carefully matched to PSB buffer (pH 7.4) supplemented with 5% DMSO (to facilitate the preparation of heme). The sample in the syringe was titrated into the cell in 19 injections (a single injection of 0.4 μl followed by 18 injections of 2.0 μl) at and the cell was stirred at 1000 rpm. The experiment was carried out at 25 °C. Data were analyzed using the MicroCal ORIGIN software (Malvern), excluding the first injection, with a “One set of sites” fitting model. A representative titration is shown (N = 2).

### Heme transfer assay

Heme transfer experiments among NEAT domains and Shp were performed following the previous study with some modifications (55). Prior to the experiments, to prepare completely heme-bound form of donor protein, the protein sample was mixed with the hemin chloride dissolved in DMSO at a molar ratio of 1: 1. The excess amount of heme or weakly bound heme was removed by loading the sample into PD-10 desalting column equilibrated with the basic assay buffer (20 mM Tris, 500 mM NaCl pH 8.0). An equal amount of heme-bound form of donor protein and heme-unbound form of acceptor protein (50 μM each) was mixed and incubated for 1 min for the heme transfer reaction to proceed. The protein mixture was separated by IMAC in a Ni-NTA agarose column equilibrated with the basic assay buffer supplemented with 20 mM Imidazole. The fraction containing the acceptor protein was collected from the flowthrough and wash fractions after adding the basic assay buffer supplemented with 20 mM Imidazole. The fraction containing the donor protein, which has His_6_tag, was collected from elution by adding the basic assay buffer supplemented with 500 mM Imidazole. The UV-visible spectra of donor protein or acceptor protein between 250 nm to 500 nm were measured using NanoDrop 1000 (ThermoFisher Scientific).

## Data availability

The coordinates and structure factors of Linker-NEAT1 and NEAT2 have been deposited in the Protein Data Bank with entry code 9W5Z and 9W5Y, respectively. All remaining data are contained within the article.

## Supporting information

This article contains supporting information.

## Conflict of interest

The authors declare that they do not have any conflicts of interest with the content of this article.
