# Supplementary material for: Structural basis for heme binding by the Shr protein from Streptococcus pyogenes
Source: J Biol Chem. 2025 Dec 5;302(1):111012. doi: 10.1016/j.jbc.2025.111012 (PMC12796733; doi:10.1016/j.jbc.2025.111012)
Supplement: Supplementary Material 1 [file mmc1.pdf]

Supplementary information for

Structural basis for heme binding by the Shr protein from *Streptococcus pyogenes*.

Kanta Seki[1], Akinobu Senoo[1], Satoru Nagatoishi[2,3], Saeko Yanaka,[1,4] Makoto Nakakido[3,5], Kouhei Tsumoto[3,5], and Jose M.M. Caaveiro[1*]

[1]Laboratory of Protein Drug Discovery, Graduate School of Pharmaceutical Sciences, Kyushu University, 3-1-1 Maidashi, Higashi-ku, Fukuoka 812-8582, Japan.

[2]Medical Device Development and Regulation Research Center, School of Engineering, The University of Tokyo, Hongo, Bunkyo-ku, Tokyo 113-0033, Japan.

[3]Department of Bioengineering, School of Engineering, The University of Tokyo, 7-3-1 Hongo, Bunkyo-ku, Tokyo 113-8656, Japan.

[4]Materials and Structures Laboratory, Institute of Integrative Research, Institute of Science Tokyo, 4259 Nagatsuta-cho, Midori-ku, Yokohama, Kanagawa 226-8503, Japan

[5]Department of Chemistry and Biotechnology, School of Engineering, The University of Tokyo, 7-3-1 Hongo, Bunkyo-ku, Tokyo 113-8656, Japan.

SUPPLEMENTARY TABLES

Table S1 | Per residue accessible surface area (ASA) and buried surface area (BSA)[a] in the interaction between Linker-NEAT1 and heme (all data is given in Å^2).

	Residue	ASA	BSA	H[b]		Residue	ASA	BSA	H[b]
**ChainA**	Lys382	89.33	30.93		**ChainB**	Lys382	87.47	33.39	
	Glu383	79.04	0.73			Glu383	78.23	0.29	
	Ser389	16.65	10.80	H_{SC}		Ser389	13.77	9.52	H_{SC}
	Met390	165.84	69.80	H_{MC}		Met390	162.32	72.89	H_{MC}
	Leu391	12.66	12.66			Leu391	11.39	11.39	
	Ala394	23.26	14.21			Ala394	24.67	15.17	
	Phe395	12.25	3.12			Phe395	13.03	2.94	
	Asn415	1.89	1.89			Asn415	0.74	0.74	
	Ala417	34.69	1.96			Ala417	35.28	1.84	
	Leu418	126.45	32.93			Leu418	121.55	31.65	
	Phe421	96.42	15.12			Phe421	98.27	12.19	
	Leu422	13.81	9.87			Leu422	14.49	11.06	
	Val470	14.43	13.57			Val470	17.66	16.57	
	Val472	29.73	26.45			Val472	26.07	23.76	
	Ala474	74.70	28.98			Ala474	75.13	31.53	
	Met475	103.01	50.62			Met475	99.21	51.75	
	Thr489	75.89	23.10			Thr489	70.66	23.25	
	Lys490	60.58	6.99			Lys490	56.89	8.17	
	Leu491	8.20	8.20			Leu491	7.42	7.42	
	Total	***1038.83***	***361.93***			***Total***	***1014.25***	***365.52***	
**HEME**		***775.30***	***512.10***		**HEME**		***775.54***	***521.91***	

[a]Accessible surface area (ASA) and buried surface area (BSA) were calculated by PISA server.

[b]H_{SC} and H_{MC} refers to a hydrogen bond formed by side chain or main chain of the indicated residue, respectively.

Table S2 | Per residue accessible surface area (ASA) and buried surface area (BSA)[a] in the interaction between Linker region and NEAT1 (all data is given in Å^2).

ChainA	Residue	ASA	BSA	H[b]	ChainB	Residue	ASA	BSA	H[b]
**Linker**	Asn318	80.90	27.86	H_{SC}	**Linker**	Asn318	82.28	0.12	
	Asp320	111.91	28.99			Asp320	114.94	17.59	
	Ser358	42.15	2.52			Asp324	70.65	2.09	
	Ala361	75.77	10.93			Lys328	64.86	3.82	
	Gly362	58.42	27.76			Ser358	38.50	16.32	
	Glu363	106.28	42.83			Gly362	56.91	14.39	
	Val364	166.80	94.30	H_{MC}		Glu363	111.82	52.41	
	Total	***642.23***	***235.19***			Val364	185.05	108.87	H_{MC}
						Total	***725.01***	***215.61***	
**NEAT1**	Lue369	121.85	31.51		**NEAT1**	Lue369	116.81	24.36	
	Thr370	107.18	61.95	H_{SC}		Thr370	103.35	19.08	
	Tyr374	14.75	9.92			Tyr374	9.53	2.21	
	Lys497	153.93	3.31			Lys497	162.25	6.51	
	Thr498	106.73	43.79			Thr498	108.64	61.05	
	Val499	38.24	28.24	H_{MC}		Val499	38.96	27.90	H_{MC}
	Thr500	95.21	37.71			Thr500	89.25	27.29	
	Lys501	153.04	11.65			Lys501	192.54	35.78	
	Total	***790.93***	***228.08***			***Total***	***821.33***	***204.18***	

[a]Accessible surface area (ASA) and buried surface area (BSA) were calculated by PISA server.

[b]H_{SC} and H_{MC} refers to a hydrogen bond formed by side chain or main chain of the indicated residue, respectively.

Table S3 | Per residue accessible surface area (ASA) and buried surface area (BSA)[a] in the interaction between NEAT2 and heme (all data is given in Å^2).

	Residue	ASA	BSA	H[b]
**ChainA**	Lys989	102.34	37.53	
	Ser996	14.41	12.17	H_{SC}
	Met997	113.89	65.29	H_{MC}
	Ser998	22.73	22.57	
	Ala1001	8.20	8.20	
	Phe1021	11.03	2.66	
	Arg1022	143.49	0.24	
	Ile1024	31.96	21.42	
	Val1026	101.76	35.28	
	Met1029	91.44	1.05	
	Gly1031	10.58	9.41	
	Tyr1032	26.11	0.62	
	Leu1033	16.38	13.69	
	Val1102	10.18	9.04	
	Val1104	23.09	23.09	
	Ile1106	29.36	19.25	
	Met1107	50.92	48.59	
	Ile1110	139.96	27.13	
	Ser1114	37.03	0.17	
	Gln1117	73.22	31.84	
	Thr1118	32.51	8.09	
	Val1119	4.85	4.85	
	Total	***1095.44***	***402.18***	
**HEME**		***773.41***	***572.99***	

	Residue	ASA	BSA	H[b]
**ChainB**	Lys989	104.76	23.59	
	Ser996	16.07	11.89	H_{SC}
	Met997	100.00	52.72	H_{MC}
	Ser998	21.08	21.08	
	Ala1001	9.36	9.36	
	Phe1021	12.75	4.37	
	Arg1022	140.44	1.22	
	Gly1023	15.40	0.33	
	Ile1024	30.55	17.52	
	Val1026	90.43	26.78	
	Met1029	65.53	3.32	
	Gly1031	9.90	9.23	
	Tyr1032	27.02	0.47	
	Leu1033	17.42	14.72	
	Val1102	15.56	12.96	
	Val1104	19.90	19.90	
	Ile1106	16.78	14.41	
	Met1107	43.51	43.51	
	Ile1110	122.58	32.95	
	Ser1111	58.74	3.01	
	Ser1114	39.46	21.81	
	Gln1117	72.72	49.02	H_{SC}
	Thr1118	30.89	8.82	
	Val1119	5.36	5.36	
	Total	***1086.21***	***408.35***	
**HEME**		***789.23***	***622.59***	

[a]Accessible surface area (ASA) and buried surface area (BSA) were calculated by PISA server.

[b]H_{SC} and H_{MC} refers to a hydrogen bond formed by side chain or main chain of the indicated residue, respectively.

Table S4 | Position of the Soret band in the heme transfer assay.

Forward transfer				Reverse transfer			
**Role**	**Construct**	**Status**	**Wavelength (nm)[a]**	**Role**	**Construct**	**Status**	**Wavelength (nm)**
Donor	Linker-NEAT1	Before	415 (ferric)	Donor	NEAT2[b]	Before	427 (ferrous)
		After	415 (ferric)			After	415 (ferric)
Acceptor	NEAT2	Before	-	Acceptor	Linker-NEAT1	Before	-
		After	412 (ferric)			After	412 (ferric)
Donor	Linker-NEAT1	Before	415 (ferric)	Donor	Shp[b]	Before	427 (ferrous)
		After	415 (ferric)			After	412 (ferric)
Acceptor	Shp	Before	-	Acceptor	Linker-NEAT1	Before	-
		After	418 (ferric)			After	412 (ferric)
Donor	NEAT2	Before	415 (ferric)	Donor	Shp[b]	Before	427 (ferrous)
		After	415 (ferric)			After	412 (ferric)
Acceptor	Shp	Before	-	Acceptor	NEAT2	Before	-
		After	418 (ferric)			After	412 (ferric)
				Donor	NEAT2[c]	Before	410 (ferric)
						After	415 (ferric)
				Acceptor	Linker-NEAT1	Before	-
						After	410 (ferric)

[a] The predominant heme species (ferric or ferrous) bound to the receptor is indicated.
[b] NEAT2 and Shp were purified as the heme-bound complex from the expression host.
[c] NEAT2 was purified as heme-free form, after which the protein was loaded with hemin chloride (ferric) before the transfer reaction.

Table S5 | Thermodynamic parameters corresponding to the interaction between heme and Shr or Shp receptors from *S. pyogenes*.

	n	ΔH (kcal/mol)	$-T\Delta S$ (kcal/mol)	ΔG (kcal/mol)	K_D (nM)
Linker-NEAT1	1.5 ± 0.1	-1.8 ± 0.2	-8.6 ± 0.3	-10.3 ± 0.5	32.0 ± 23.4
NEAT2	0.9 ± 0.1	-2.8 ± 0	-8.2 ± 0.7	-11.0 ± 0.7	12.7 ± 10.3
Shp	1.1 ± 0	-2.2 ± 0.1	n.d.	n.d.	<0.81*

** Since the Wiseman parameter (c-value) of the titration exceeded the threshold of 1,000 (almost vertical transition), the determined value of the dissociation constant may be considered the lower limit of affinity, but it is not a reliable value to calculate the change of free energy and change of entropy.*

Table S6 | Structures of NEAT domains from different gram-positive bacteria in the PDB.

Species name	Protein	Heme interacting residues	PDB ID	Reference Number
Staphylococcus aureus	IsdA NEAT	Tyr166	2ITF	39
	IsdB NEAT2	Met362, Tyr440	3RTL	40
	IsdC NEAT	Tyr132	2O6P	41
	IsdH NEAT3	Tyr642	2Z6F	42
Bacillus anthracis	IsdX1 NEAT	Tyr136	3SIK	13
	IsdX2 NEAT5	Tyr108	4H8P	12
Listeria monocytogenes	Hbp2 NEAT2	Tyr280	4MYP	14
Streptococcus pyogenes	Shp	Met66, Met153	2Q7A	43
	Shr NEAT1	Met390, Met475	9W5Z	This study
	Shr NEAT2	Met997, Met1107	9W5Y	This study

SUPPLEMENTARY FIGURES

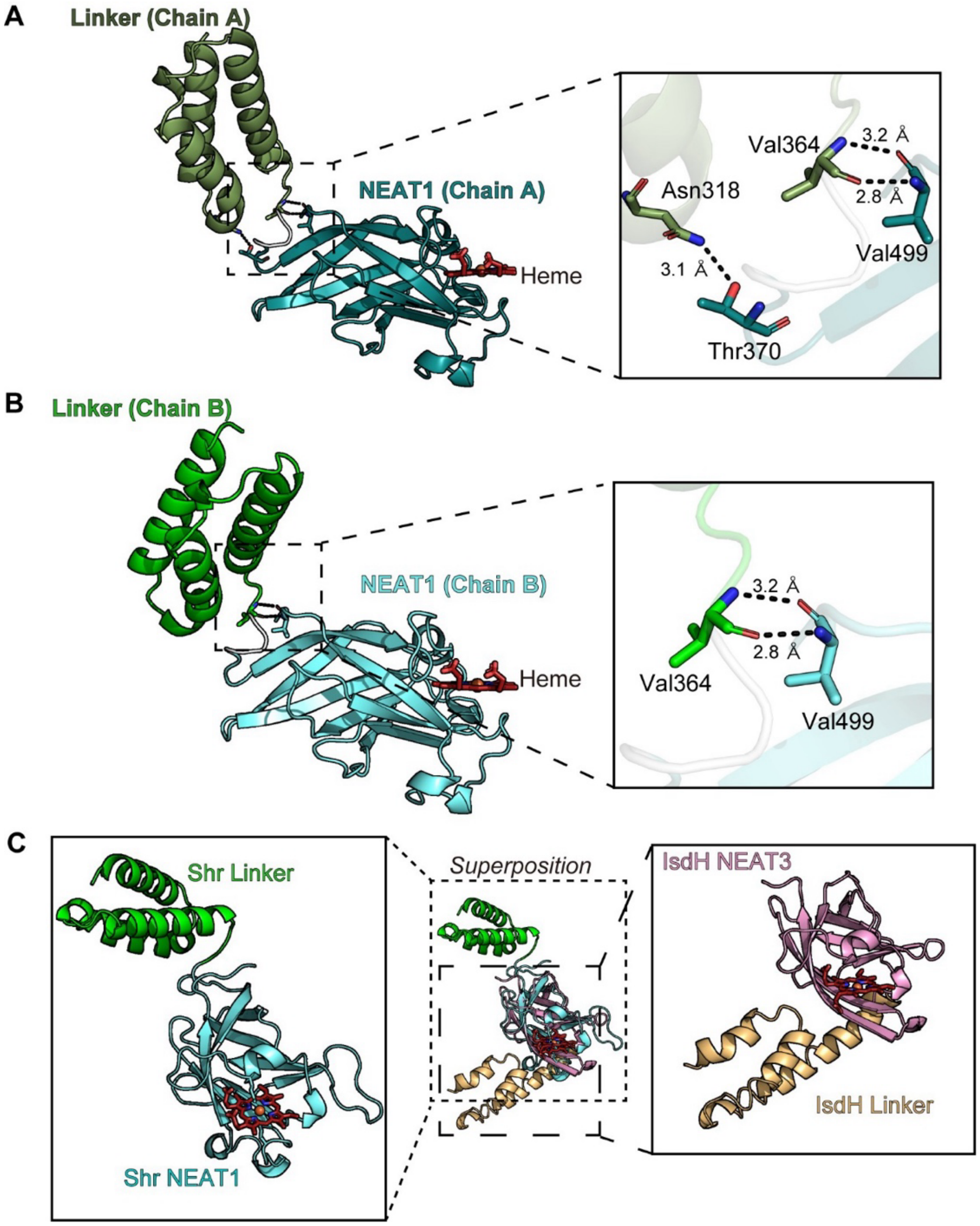

Figure S1 | Linker region of NEAT1. (A), **(B)** The intramolecular interactions between residues of the linker region and the NEAT1 domain. The left panel shows the whole structure of either chain A or B in the crystallographic asymmetric unit of Linker-NEAT1. The residues forming hydrogen bonds are shown in the close-up image on the right. **(C)** Comparison of the structure of the Shr Linker-NEAT1 with that of IsdH Linker-NEAT3 (PDB ID: 7W81). The superposition of both structures aligned by their NEAT domains is shown in the middle panel. The individual structures of Shr Linker-NEAT1 and IsdH Linker-NEAT3 are depicted on the left and right panels, respectively. The length of the three helixes were different in both structures. Besides, the relative position of the linker region with respect to the NEAT domain was different in the two proteins.

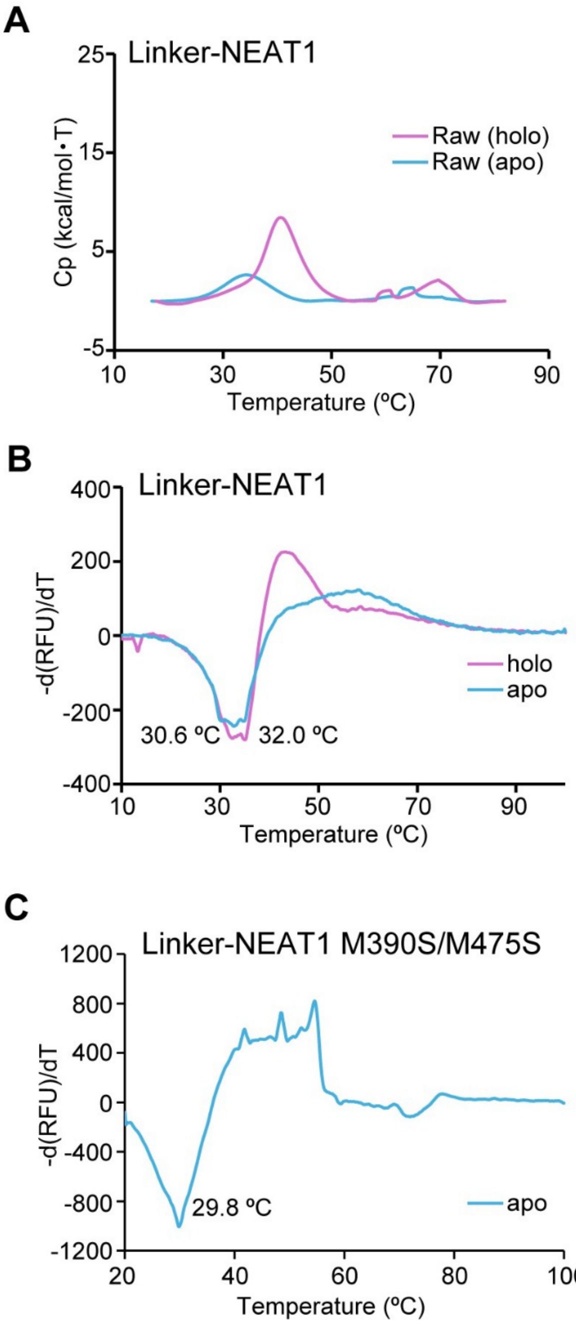

Figure S2 | Thermostability of Linker-NEAT1 and NEAT2. (A) Raw data from DSC measurement. These data were used for the fitting curve shown in Figure 2 and Figure 4 of the manuscript. **(B)** Stability of WT proteins (with and without heme) and **(C)** double mutants determined by DSF. Derivatives of relative fluorescent unit (RFU) over derivatives of temperature (-d(RFU)/dT) are shown.

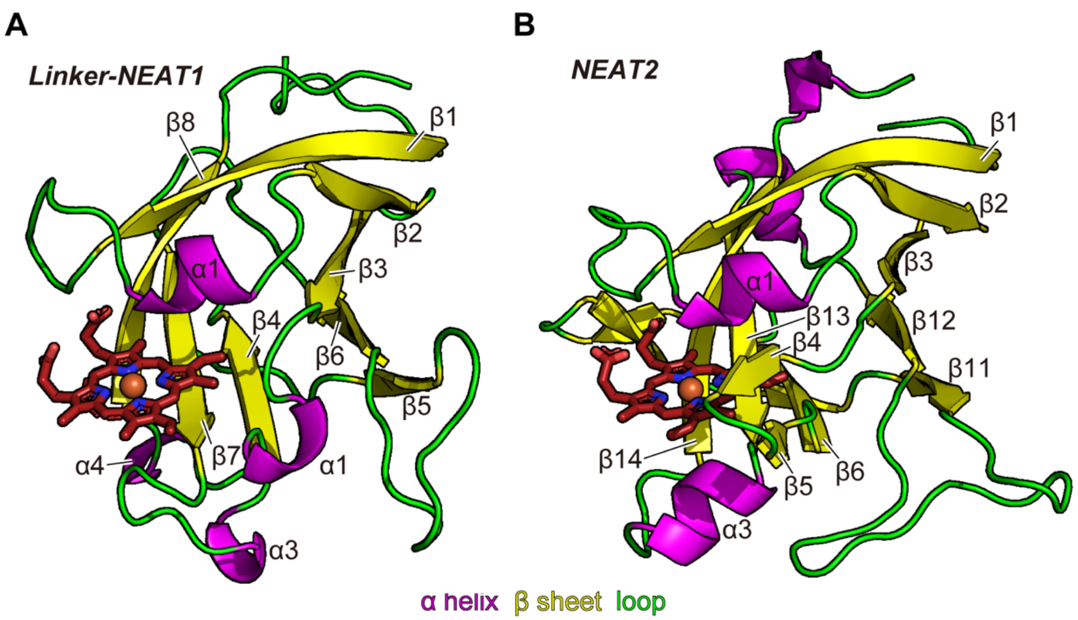

Figure S3 | Comparison of NEAT1 and NEAT2 of Shr. Structure of NEAT1 **(A)** and structure of NEAT2 **(B)** in which α-helices, β-sheet and loop regions in the structure are colored in magenta, yellow and green, respectively. The following elements, α1, β1-β2-β3 and β4-β5-β6-β7-β8 of NEAT1 correspond to α1, β1-β2-β3 and β6-β11-β12-β13-β14 of NEAT2. These secondary elements are common in both NEAT domains. In NEAT2, β4-β5 and α3 specifically contribute to the formation of a deeper heme-binding pocket.

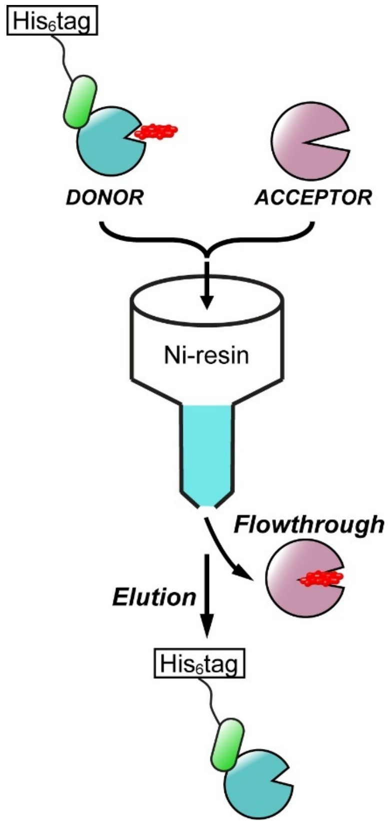

Figure S4 | Schematic illustration of heme transfer experiments. In our experiments, all donor proteins contained a His_6tag, whereas the acceptor protein did not. After incubation of donor and acceptor, the proteins were separated by Immobilized Metal Affinity Chromatography (IMAC) in a Ni-NTA resin. The absorbance spectrum of the flowthrough and elution fractions were subsequently examined to evaluate the extent of heme transfer.

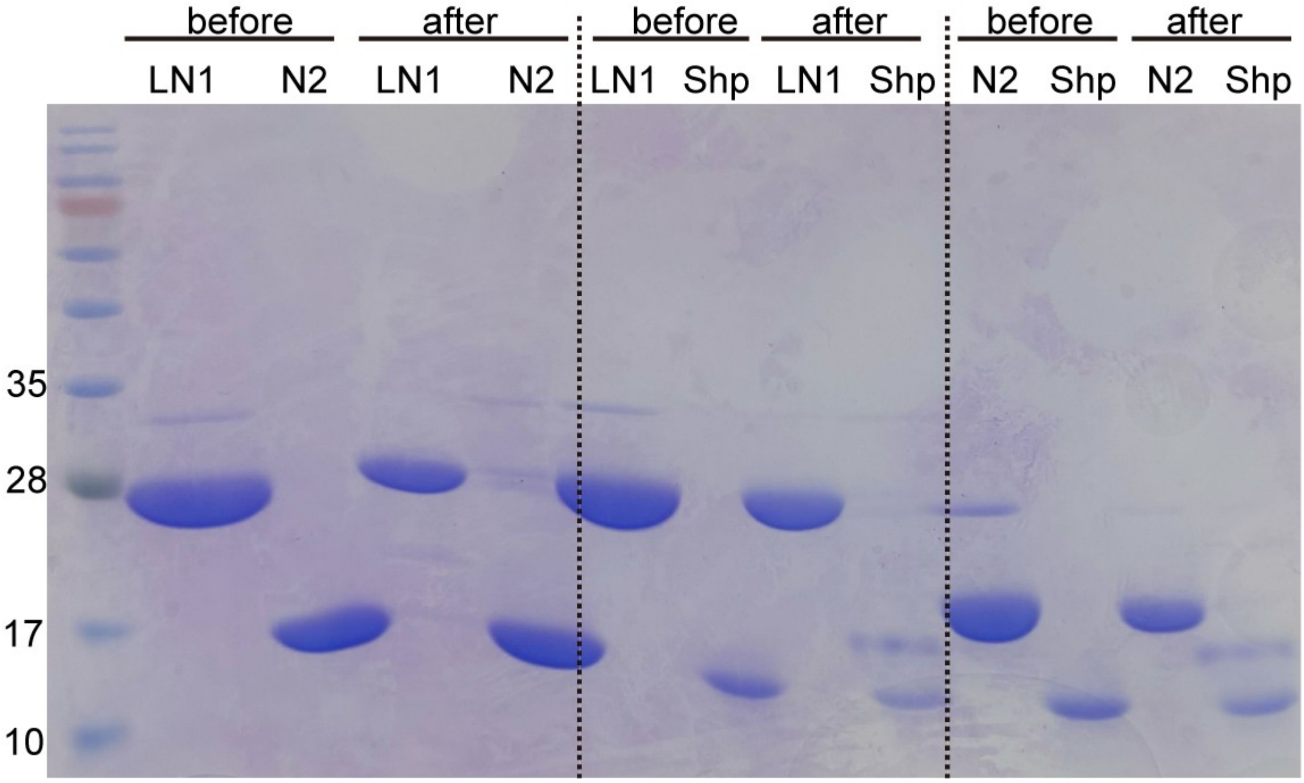

Figure S5 | Confirmation of separation of donor from acceptor proteins in the heme transfer assay. The precise separation of donor or acceptor proteins after heme transfer reaction was confirmed using SDS-PAGE with Coomassie Brilliant Blue stain. LN1 and N2 represent Linker-NEAT1 and NEAT2, respectively. Samples before or after heme transfer were examined. In each pair of samples, donor and acceptor protein wer loaded on the left and right lanes, respectively. The expected molecular mass are following: LN1, 25.0 kDa; N2 (donor), 19.7 kDa; N2 (acceptor), 17.9 kDa; and Shp, 16.5 kDa.

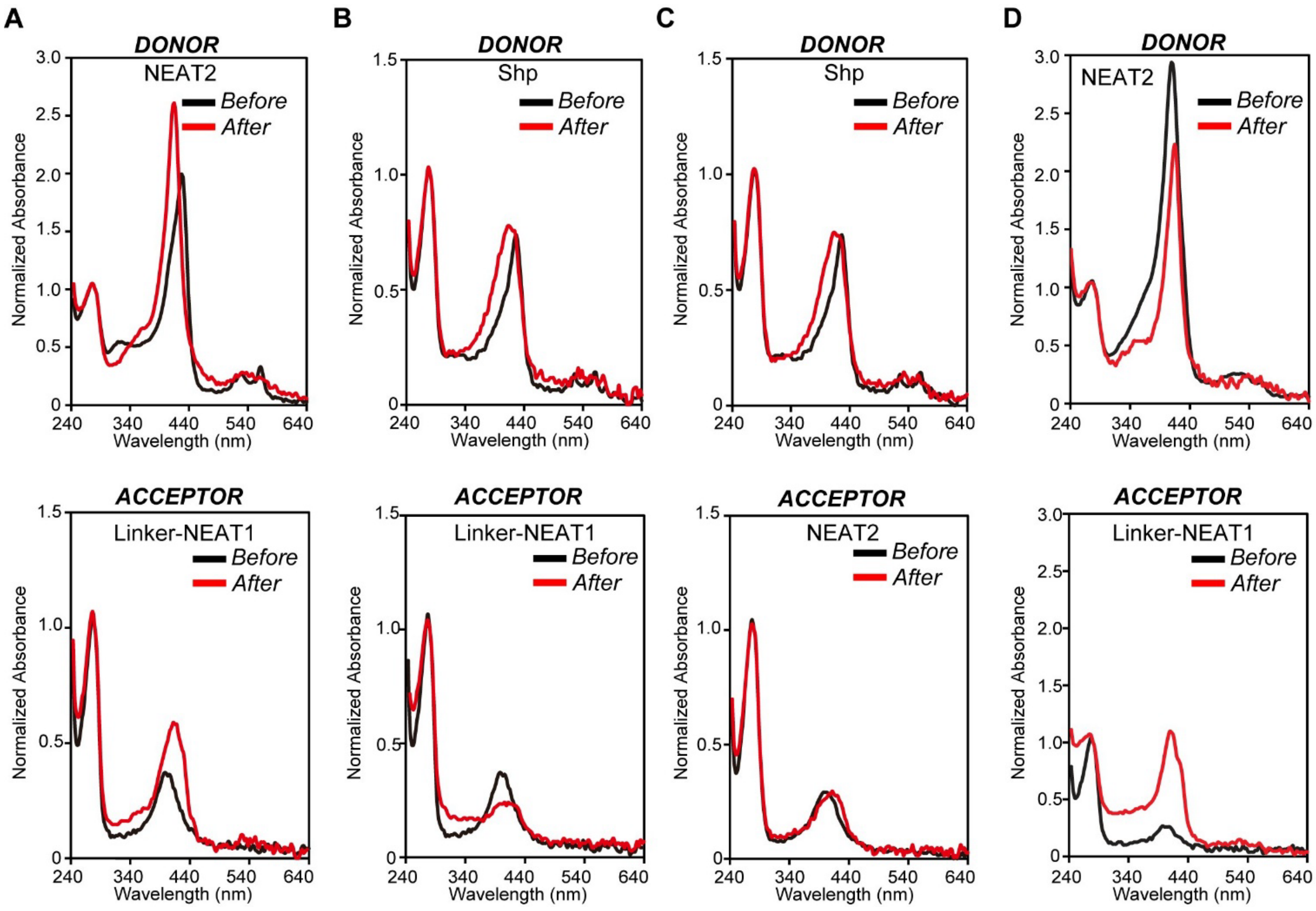

Figure S6 | Heme transfer assay among Linker-NEAT1, NEAT2 and Shp in the reverse direction compared to that in Figure 5 of the manuscript. (A) Heme transfer experiment from NEAT2 to Linker-NEAT1, **(B)** from Shp to Linker-NEAT1, and (**C)** from Shp to NEAT2. In these experiments, the endogenous heme from E. coli is bound to the donor proteins as a mixture of predominantly ferrous heme. **(D)** Heme transfer experiment from NEAT2 to Linker-NEAT1, in which the donor (NEAT2) was prepared by adding ferric heme (hemin chloride) to the heme-free form, so a direct comparison with the forward experiment form Figure 6 could be established. In each panel, upper and lower panels show the absorbance spectra of heme donors or heme acceptors. The black and red solid lines show the spectra before and after the transfer reaction, respectively. After the reaction the Soret band of heme bound to all proteins appeared at ~415 nm, corresponding to the ferric form (see Table S6).

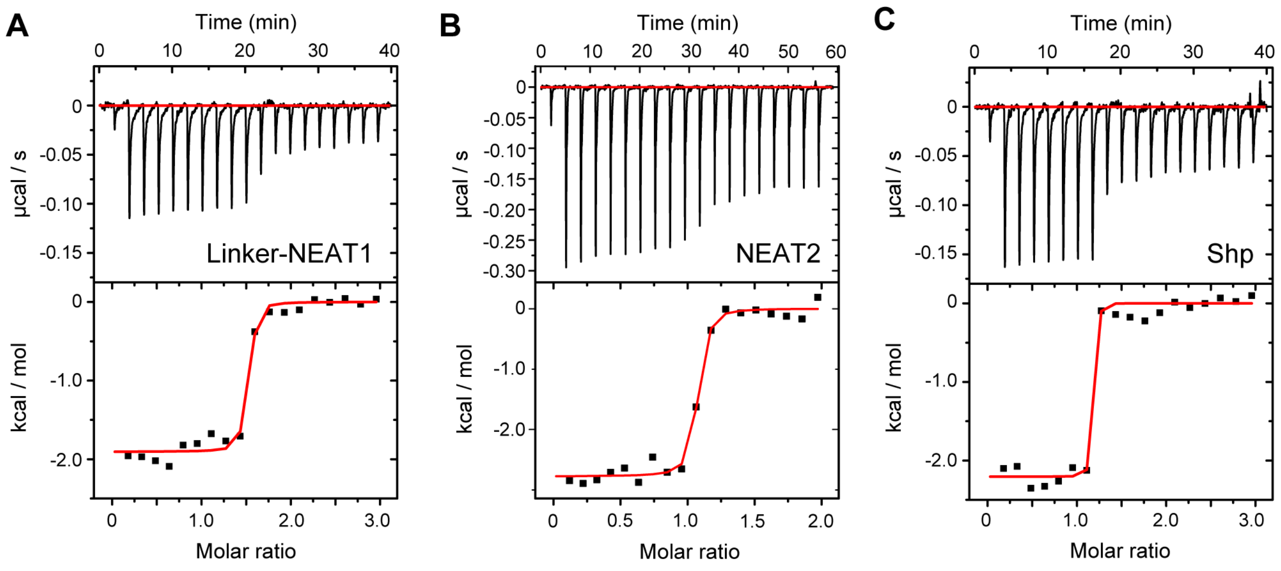

Figure S7 | Binding of heme to receptors as monitored by ITC. (A)~(C) Interaction between heme and **(A)** Linker-NEAT1, **(B)** NEAT2, or **(C)** Shp. Experiments were carried out with protein and heme concentrations of 20 and 300 μM, respectively. The top panels correspond to the titration kinetics, and the bottom panels correspond to the integrated binding isotherms. The binding enthalpy (Δ*H*) and the dissociation constant (K_D) were determined by nonlinear regression of the integrated data to a one- site binding model using the Origin software. (N =2)
